# The multimorbidity association of metabolic syndrome and depression on type 2 diabetes: a general population cohort study in Southwest China

**DOI:** 10.3389/fendo.2024.1399859

**Published:** 2024-07-05

**Authors:** Yulan Cai, Shiyu Zhou, Shangheng Fan, Yan Yang, Kunming Tian, Lei Luo, Renli Deng, Xingyu Dai, Yiying Wang, Minglan Zhu, Tao Liu

**Affiliations:** ^1^ Department of Endocrinology and Metabolism, The Second Affiliated Hospital of Zunyi Medical University, Zunyi, Guizhou, China; ^2^ Department of Nursing, Affiliated Hospital of Zunyi Medical University, Zunyi, Guizhou, China; ^3^ Department of Preventive Medicine, School of Public Health, Zunyi Medical University, Zunyi, Guizhou, China; ^4^ School of Clinical Medicine, Zunyi Medical University, Zunyi, Guizhou, China; ^5^ Department of Chronic Disease Prevention and Control, Guizhou Disease Prevention and Control, Guiyang, Guizhou, China

**Keywords:** type 2 diabetes, metabolic syndrome, depression, complication, multimorbidity, cohort study

## Abstract

**Background:**

Metabolic syndrome(MetS) and depression are independently associated with type 2 diabetes (T2DM) risk. However, little is known about the combined effect of MetS and depression on the risk of T2DM. The present study aims to prospectively explore the impact of MetS and depression on T2DM susceptibility among the Chinese general population.

**Methods:**

6489 general population without T2DM adults in Southwest China were recruited from 2010 to 2012. Depression and MetS were prospectively assessed using a 9-item Patient Health Questionnaire(PHQ-9) and Guideline for the prevention and treatment of type 2 diabetes mellitus in China (2020 edition) (CDS2020) during 2016–2020, respectively. Modified Poisson regression models were conducted to estimate relative risk(RR) and 95% confidence intervals (95%CI) for independent and combined associations of MetS and depression with an incidence of T2DM.

**Results:**

During a median follow-up of 6.6 years, 678 cases of T2DM were documented. Individuals with MetS were 1.33 times more likely to develop T2DM than those without MetS. The corresponding RR(95%CI) for depression with no depression was 1.45(1.22–1.72). Notably, compared with no MetS or depression, the multivariate-adjusted RR for a combined effect of MetS and depression on the risk of T2DM was 2.11(1.39–3.22). Moreover, an increased risk of T2DM was more apparent in those ≥ 60 years, males, and overweight.

**Conclusions:**

Individuals with multimorbidity of MetS and depression are at a higher risk of T2DM compared with those with no MetS or depression.

## Introduction

Diabetes mellitus (DM) is one of the worldwide well-recognized and uncontrollable common metabolic diseases, with 9.3% (463 million) of the global prevalence, 90% of which is type 2 diabetes mellitus (T2DM) (1, 2). The highest number of T2DM exists in China, affecting 116 million humans (3, 4). Hence, T2DM poses a severe threat and heavy economic burden to the health of the Chinese population (5). Regretfully, identified physical inactivity, genetic susceptibility, and diet habits fail to effectively and fully explain the etiology of T2DM. Notably, the complex interaction regarding different pathogenic factors also plays a significant role in the etiology of T2DM, which may present another perspective for uncovering the initiation of T2DM (6). Given that mental illness and metabolic disorder are both closely associated with the risk of T2DM (7–10). Therefore, a combined disorder of psychological disorders and metabolic disorders is likely to have a potential effect on the occurrence of T2DM.

Individuals often suffer from multiple chronic diseases at the same time, which is called multimorbidity (11). The prevalence of multimorbidity is continuously increasing, generating adverse threats to human health. Therefore, it is meaningful to dissect the complex etiology of chronic non-communicable diseases from the perspective of multimorbidity. MetS is a pathological condition including insulin resistance, abdominal obesity, hyperlipidemia, and hypertension, affecting 20–25% of adults worldwide (12, 13). MetS is consistently and independently associated with an increased risk of DM, especially for T2DM, among general population settings. Emerging evidence indicated that depression, another crucial healthcare burden, contributes to increased mortality and a panel of severe metabolic complications (14, 15). Interestingly, the population suffering from depression and using antidepressants by altering the uptake and regulation of glucose were all more prone to T2DM (16, 17).

Evidence indicates that there is an inner link between depression and MetS (18). Significantly, the above notion was strengthened by the evidence that depression is a pathogenic factor for MetS (19). Moreover, insulin resistance commonly occurs along with the occurrence of depression and implicates the progression of depression (20). The two diseases often cluster in pairs and closely interact with each other. However, the combined effect of these risk markers on the risk of T2DM remains unknown. Ample evidence has indicated that many diseases or pathological statuses could synergistically promote the incidence of T2DM. A study found that the cumulative effect of obesity and MetS significantly links to a raised incidence of T2DM (21). Furthermore, patients with depressive symptoms and poor sleep quality had lower T2DM-related quality of life compared with those who had depression or poor sleep quality (22). In addition, insulin resistance is a common important characteristic of both depression and MetS and also functions as one critical pathogenesis of T2DM (23). Thus, it is seemed to be a synergistic interaction between depression and MetS and other T2DM-related risk factor to boost increased T2DM risk.

Given the closed association of both MetS and depression with the pathogenesis of T2DM, we hypothesized that Mets and depression could be synergistically associated with T2DM in the general population. Therefore, we evaluated the combined association of MetS and depression with the risk of T2DM based on the Guizhou natural population cohort study. Our results could provide scientific evidence for preventing T2DM incidence in the population with multimorbidity of metabolic disease and mental disorders.

## Methods

### Study population

**Figure 1:**
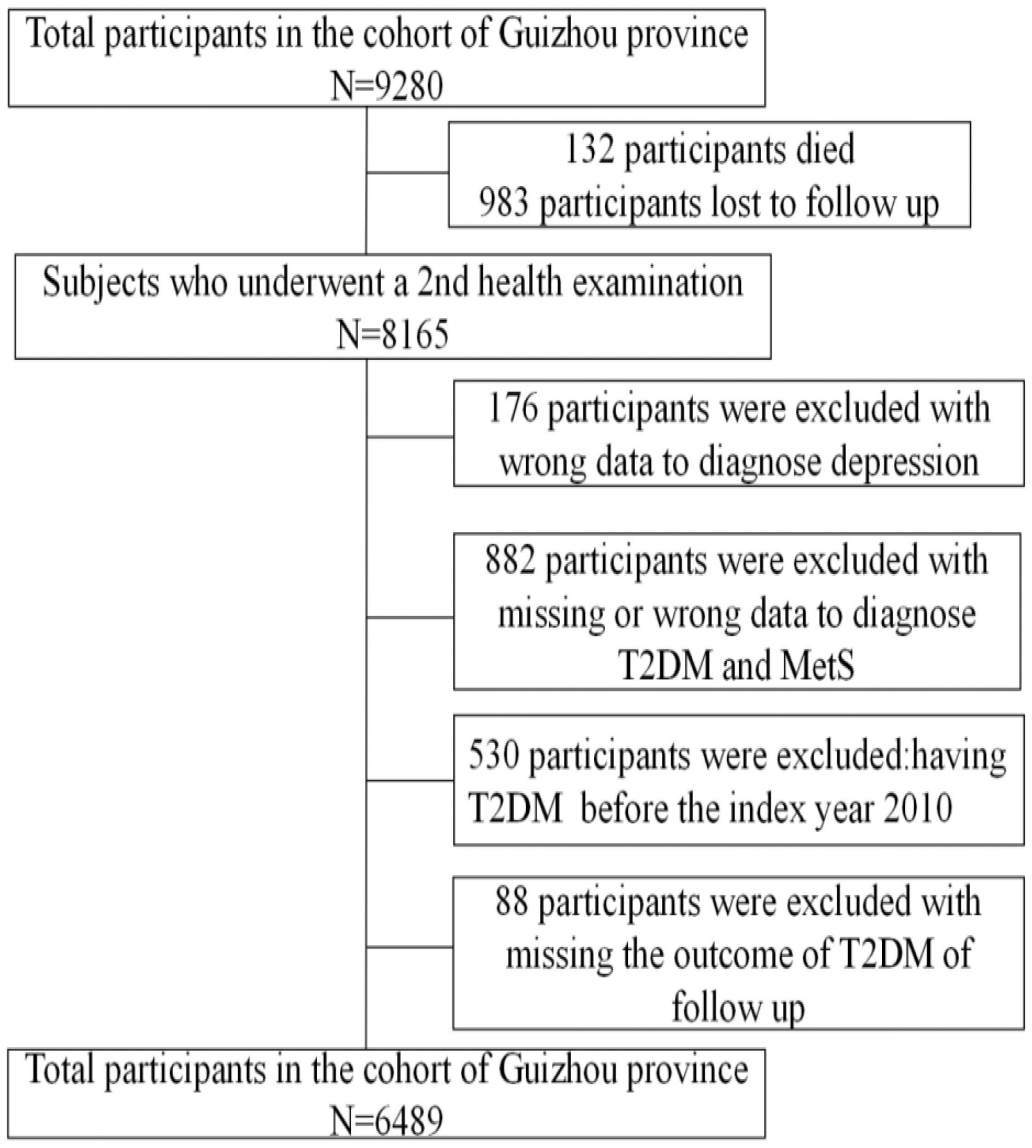
Flowchart of the study sample.

The Guizhou natural population cohort study comprised a representative sample of 9280 participants aged ≥18 years. The participants were recruited using multistage proportional stratified cluster sampling from 48 townships in 12 districts of Guizhou province between October 2010 and August 2012. A total of 8,165 study participants completed at least one follow-up visit in this 10-year follow-up study. We excluded participants who were diagnosed with T2DM at baseline (n = 530), missing outcome of T2DM at the follow-up (n = 88), and missing or wrong data at baseline (n = 1058) (**Figure 1**). After the above careful screening, 6489 remaining participants were eligible for our study. We obtained the approval of the institutional Review Committee of the Guizhou Center for Disease Control and Prevention (No.S2017–02) to implement this study. All participants also signed written informed consent forms.

### Measurement of blood biochemistry markers and lifestyle

Participants were instructed to fast overnight at least 8-12 hours before blood specimen collection. In a qualified central laboratory, trained professionals measured triglycerides (TG) and high-density lipoprotein cholesterol (HDL-C). Additionally, participants were given 75 g of glucose to perform a 2-h oral glucose tolerance test (OGTT) to test fasting blood glucose (FPG) and 2-hour postprandial blood glucose (2h PG). The assessment of sociodemographic factors (age, sex, region, education level, and marital status), anthropometric measures (weight and height), medication history and family history of diseases (T2DM, Hypertension), behavioral risk factors (smoking and alcohol consumption), dietary intakes (The daily intake of oil and salt was calculated by asking ‘how many kilos of oil/salt do you usually consume in a month’ through inquiry), level of physical activity, mental health and death information were obtained via face-to-face interviews. Blood pressure was documented with the average value of three repeated measurements using the same model electronic sphygmomanometer. Hypertension was defined by the Seventh Report of the Joint National Committee on Prevention, Detection, Evaluation, and Treatment of High Blood Pressure (JNC 7) as follows: (1) self-reported hypertension or use of hypertension medications; and (2) systolic blood pressure ≥140 mmHg and diastolic blood pressure ≥90 mmHg (24).

### Ascertainment of outcomes

T2DM was the endpoint of the study. The T2DM patients were determined as T2DM according to self-reported physician-diagnosed diabetes or use of hypoglycemic agents or blood glucose examinations. The diagnostic criteria of the American Diabetes Association (ADA, 2019), T2DM is defined as: 1) a self-reported previous diagnosis by health professionals, or 2) fasting blood glucose ≥7.0 mmol/L (126 mg/dL), or 3) 2-hour postprandial blood glucose ≥11.1 mmol/L (200 mg/dL), or 4) Hemoglobin A1c (HbA1c) concentration ≥6.5% (25).

### Assessment of depression and MetS

9-item Patient Health Questionnaire (PHQ-9), a brief self-assessment of depressive symptoms with high accuracy, reliability, and validity, has been verified by structured diagnostic interviews conducted by mental health professionals, widely used to define depression (26–28). Participants rate nine depressive symptoms and their frequency/duration over the previous two weeks. PHQ-9 is computed by summing the scores of 9 symptom items (range, 0–27). In our study, the subjects who were diagnosed with depression according to PHQ ≥5 points.

MetS was assessed according to the Guideline for the prevention and treatment of type 2 diabetes mellitus in China (CDS2020) (29). The diagnostic criteria of MetS are as follows: ① Abdominal obesity: male waist circumference ≥90 cm, female waist circumference ≥85 cm; ② Hyperglycemia: fasting blood glucose ≥6.1 mmol/L or 2-hour postprandial blood glucose ≥7.8 mmol/L and diabetes has been diagnosed and treated; ③ Hypertension: blood pressure ≥130/85 mmHg and hypertension has been confirmed and treated; ④ Fasting triglyceride ≥1.70 mmol/L; ⑤ Fasting HDL-C < l.04 mmol/L. Adult Treatment Panel III (ATP III 2005) for MetS in sensitivity analysis: (1) Asian male waist ≥90 cm, Asian female waist ≥80 cm; (2) TG ≥ 1.7 mmol/L, or have received corresponding treatment; (3) Male HDL-C < 1.03 mmol/L, female HDL-C < 1.29 mmol/L, or have received corresponding treatment; (4) Blood pressure ≥130/85 mmHg and have been diagnosed hypertension and receive corresponding treatment; (5) FPG ≥5.6 mmol/L, or those who have been diagnosed T2DM and treated (30). Those who meet three items or more can be diagnosed MetS based on CDS2020 and ATP III, respectively.

### Covariates

The covariates adjusted in regression models were based on previous studies regarding the relationship of MetS or depression with T2DM and the potential biological mechanisms. Covariates listed in our study include age, sex (male, female), region (urban or rural), ethnicity (the Han nationality or other), marital status (married or other), and education level (no formal school, primary, middle school, high school, college/university or more), smoking status (every day, sometime or never), excessive drinking status (yes or no), physical activity (never, 1–2 days per week, ≥3 days per week), oil intake (>25g/d or ≤25g/d), salt intake (>6g/d or ≤6g/d), family history of diabetes (yes or no), and body mass index (BMI). BMI was measured by weight/height (kg/m^2^). According to the 2016 Dietary Guidelines for Chinese Residents, excessive alcohol consumption was defined as men: > 25 g/day and women: > 15 g/day (31).

### Statistical analysis

We investigated the joint associations of MetS and depression with an incidence of T2DM. Participants were classified into four categories: no depression or MetS, depression only, MetS only, MetS together with depression, and those with no MetS or depression were used as a reference group. The mean standard deviation describes continuous numerical variables ( $\bar{\text{x}}$ ± sd), and classified variables are expressed as n (%). The statistical differences among the four groups at baseline were analyzed using one-way ANOVA, Kruskal Wallis, or chi-square test as appropriate. A modified Poisson regression model was used to examine the independent and synergistic association of MetS and depression with T2DM by calculating relative risk (RR) and 95% confidence interval (CI). Stratified analysis was conducted according to age, sex, and BMI to explore whether specific factors change correlation. The sets of covariates were adjusted: Model 1 consisted of age and sex; Model 2 consisted of Model 1 plus region, ethnicity, marital status, and education level; Model 3 consisted of Model 2 together with smoke now status, physical activity, excessive drinking status, oil intake, salt intake and family history of diabetes, BMI. Sensitivity analysis was performed after redefining MetS according to the criteria of ATPIII. All analyses were performed using SPSS 25.0 and R3.6.3, and statistical significance was based on a 2-sided test at the 0.05 significance level.

## Results

### Baseline characteristics of participants

The baseline characteristics in different groups are presented in **Table 1**. Participants have the highest BMI of 26.2 ± 3.68kg/m^2^ in the MetS and depression group. Individuals are more often women, the Han nationality across the four groups. No MetS or depression, and MetS only were more likely to live in rural. Participants in the groups of MetS only and MetS comorbidity with depression were older than those in the group of no MetS or depression. Overall, 6489 individuals were tracked during the 10-year follow-up, and 678 new cases of incident T2DM were documented.

**Table 1:** The baseline characteristics of the participants.

Character	Total (n=6489)	No MetS or depression (n=5097)	Depression only (n=344)	MetS Only (n=979)	MetS and depression (n=69)	P value
T2DM						<0.001
Yes	678 (10.5)	464 (9.1)	38 (11.0)	159 (16.2)	17 (24.6)	
No	5811 (89.5)	4633 (90.9)	306 (89.0)	820 (83.8)	52 (75.4)	
**Age (years)**	43.4 ± 14.96	42.2 ± 14.85	44.4 ± 14.34	49.2 ± 14.45	49.1 ± 12.37	<0.001
**BMI (kg/m^2^)**	22.8 ± 3.31	22.3 ± 2.89	22.0 ± 2.48	25.5 ± 4.02	26.2 ± 3.68	<0.001
Sex n (%)						0.003
Male	3080 (47.5)	2437 (47.8)	139 (40.4)	481 (49.1)	23 (33.3)	
Female	3409 (52.5)	2660 (52.2)	205 (59.6)	498 (50.9)	46 (66.7)	
Region n (%)						<0.001
Urban	2368 (36.5)	1904 (37.4)	172 (50.0)	254 (25.9)	38 (55.1)	
Rural	4121 (63.5)	3193 (62.6)	172 (50.0)	725 (74.1)	31 (44.9)	
Ethnicity n (%)						0.001
The Han nationality	3844 (59.2)	2964 (58.2)	224 (65.1)	604 (61.7)	52 (75.4)	
Other	2645 (40.8)	2133 (41.8)	120 (34.9)	375 (38.3)	17 (24.6)	
Education level n (%)						<0.001
No formal school	2268 (35.0)	1767 (34.7)	162 (47.1)	306 (31.3)	33 (47.8)	
Primary	1349 (20.8)	1082 (21.2)	57 (16.6)	197 (20.1)	13 (18.8)	
Middle school	1974 (30.4)	1552 (30.5)	86 (25.0)	318 (32.5)	18 (26.1)	
High school	587 (9.0)	456 (8.9)	21 (6.1)	106 (10.8)	4 (5.8)	
College/university or more	311 (4.8)	240 (4.7)	18 (5.2)	52 (5.3)	1 (1.4)	
Marital status n (%)						0.728
Married	5208 (80.3)	4094 (80.3)	281 (81.7)	776 (79.3)	57 (82.6)	
Other	1281 (19.7)	1003 (19.7)	63 (18.3)	203 (20.7)	12 (17.4)	
Smoke now n (%)						0.044
Everyday	1645 (25.4)	1272 (25.0)	76 (22.1)	285 (29.1)	12 (17.4)	
Sometime	195 (3.0)	152 (3.0)	9 (2.6)	32 (3.3)	2 (2.9)	
Never	4649 (71.6)	3673 (72.0)	259 (75.3)	662 (67.6)	55 (79.7)	
Excessive drinking n (%)						0.004
No	5837 (90.0)	4606 (90.4)	311 (90.4)	853 (87.1)	67 (97.1)	
Yes	652 (10.0)	491 (9.6)	33 (9.6)	126 (12.9)	2 (2.9)	
Physical activity n (%)						0.005
Never	5922 (91.3)	4683 (91.9)	313 (91.0)	864 (88.3)	62 (89.9)	
1–2 days per week	162 (2.5)	116 (2.3)	14 (4.1)	30 (3.1)	2 (2.9)	
≥3 days per week	405 (6.2)	298 (5.8)	17 (4.9)	85 (8.7)	5 (7.2)	
Family history of diabetes n (%)						0.006
No	6399 (98.6)	5039 (98.9)	334 (97.1)	959 (98.0)	67 (97.1)	
Yes	90 (1.4)	58 (11.1)	10 (2.9)	20 (2.0)	2 (2.9)	
Salt intake>6g/day n (%)						0.348
No	1924 (29.7)	1515 (29.7)	111 (32.3)	282 (28.8)	16 (23.2)	
Yes	4565 (70.3)	3582 (70.3)	233 (67.7)	697 (71.2)	53 (76.8)	
Oil intake>25g/day n (%)						0.023
No	1916 (29.5)	1549 (30.4)	97 (28.2)	250 (25.5)	20 (29.0)	
Yes	4573 (70.5)	3548 (69.6)	247 (71.8)	729 (74.5)	49 (71.0)	

### Independent and synergistic effect of MetS and depression on T2DM

As shown in **Table 2**, the incidence rate of T2DM was 13.32 and 16.79 in the depression and MetS groups. We explored the independent effect of depression or MetS on the occurrence of T2DM. Statistically significant results were observed between depression and no depression after adjustment for covariates 1.31(1.01–1.68). Similarly, compared to participants with no MetS, the incidence was approximately two times stronger predictor of T2DM (RR, 1.65, 95% CI, 1.40–1.94) for MetS patients. After further adjustment for region, ethnicity, marital status, education level, smoke-now status, excessive drinking status, physical activity, oil intake, salt intake, family history of diabetes, and BMI, the relative risk remained statistically significant (RR, 1.45, 95% CI, 1.22–1.72).

**Table 2:** Independent association of MetS or depression with risk of incident type 2 diabetes.

Subgroup	Case/Total	Incidence rate (%)	Model 1	Model 2	Model 3
Depression					
No	623/6076	10.25	1.00 (ref.)	1.00 (ref.)	1.00 (ref.)
Yes	55/413	13.32	1.27 (0.99–1.64)	1.31 (1.01–1.68)	1.33 (1.03–1.71)
Mets					
No	502/5441	9.23	1.00 (ref.)	1.00 (ref.)	1.00 (ref.)
Yes	176/1048	16.79	1.65 (1.40–1.94)	1.61 (1.37–1.90)	1.45 (1.22–1.72)

RR, relative risk; CI, confidence interval.

Model 1: adjusted for age and sex.

Model 2: model 1 plus region, ethnicity, marital status, and education level.

Model 3: model 2 plus smoke now status, excessive drinking status, physical activity, oil intake, salt intake, family history of diabetes, and BMI.

It is noteworthy that MetS combined with depression, as a multimorbidity status, was synergistically linked to the argued incidence of T2DM, as showed by RR 2.49(1.64,3.79), and the risk was higher than that of MetS only 1.61(1.36,1.91) or depression only 1.18(0.87,1.61). After adjustment for model 1 + current smoking status, physical activity, excessive drinking status, oil intake, salt intake, family history of diabetes, and BMI, the RR is 2.11(1.39,3.22). The P value remains significant, as shown in **Table 3**.

**Table 3:** Combined effect of MetS and depression status and risk of incident type 2 diabetes.

MetS or depression status	Case/Total	Incidence rate (%)	Model 1	Model 2	Model 3
No MetS or depression	464/5097	9.10	1.00 (ref.)	1.00 (ref.)	1.00 (ref.)
Depression only	38/344	11.05	1.18 (0.87,1.61)	1.20 (0.88,1.63)	1.23 (0.90,1.67)
MetS only	159/979	16.24	1.61 (1.36,1.91)	1.57 (1.32,1.86)	1.33 (1.11,1.59)
MetS and depression	17/69	24.64	2.49 (1.64,3.79)	2.51 (1.65,3.81)	2.11 (1.39,3.22)

RR, relative risk; CI, confidence interval.

Model 1: adjusted for age and sex.

Model 2: model 1 plus region, ethnicity, marital status, and education level.

Model 3: model 2 plus smoke now status, excessive drinking status, physical activity, oil intake, salt intake, family history of diabetes, and BMI.

### Subgroup analysis and effect modification

**Figure 2:**
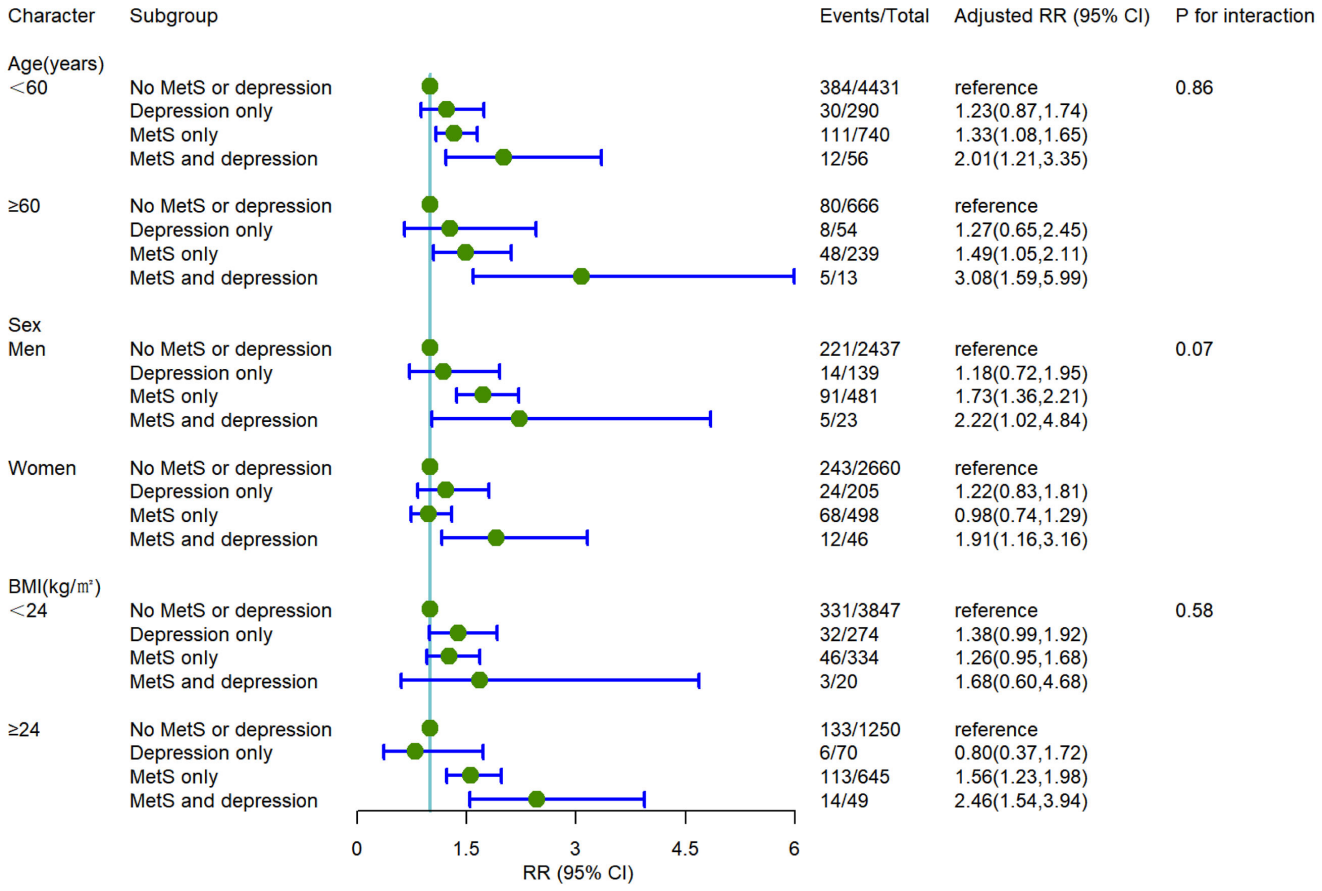
The incident risk of T2DM associated with MetS and depression by age, sex, and BMI. All analyses were adjusted for model 3 covariates. RR, relative risk; CI, confidence interval; BMI, body mass index.

The baseline population was stratified by age (< 60 years old, ≥60 years old), sex (male, female), and BMI (≥ 24kg/m^2^, < 24kg/m^2^) to explore the modifying effect of the above significant characteristics on the association between depression combined with MetS and the incidence of T2DM. Compared with the population with no MetS or depression, individuals with MetS combined with depression had a significantly higher incidence of T2DM in the subgroups of age ≥60 years [RR (95%CI) 3.08(1.59,5.99)], male [RR (95%CI) 2.22 (1.02,4.84)], BMI ≥ 24kg/m^2^ [RR (95%CI) 2.46 (1.54,3.94)] after fully adjusting potential confounding factors. The association for MetS combined with depression with the risk of T2DM is more evident in those aged 60 years or older, males, and the overweight population. The subgroup analysis is shown in **Figure 2**.

### Sensitivity analysis

The relationship between MetS combined with depression and T2DM after redefined criteria of MetS was also analyzed according to the diagnostic criteria of ATP III. Compared with individuals with no MetS or depression, the RR (95%CI) value of people with MetS and depression were [2.17 (1.44–3.28) and 1.84 (1.22–2.78)] after adjusting Model 1 and Model 3. **Table 4** depicts the sensitivity analysis.

**Table 4:** Sensitivity analysis using different definitions of MetS.

Subgroup	Case/Total	Incidence rate (%)	Model 1	Model 2	Model 3
No MetS or depression	418/4771	8.76	1.00 (ref.)	1.00 (ref.)	1.00 (ref.)
Depression only	36/318	11.32	1.26 (0.92–1.73)	1.28 (0.92–1.76)	1.31 (0.95–1.79)
MetS only	205/1305	15.71	1.67 (1.42–1.95)	1.61 (1.37–1.89)	1.38 (1.16–1.64)
MetS and depression	19/95	20.00	2.17 (1.44–3.28)	2.15 (1.42–3.23)	1.84 (1.22–2.78)

RR, relative risk; CI, confidence interval.

Model 1: adjusted for age and sex.

Model 2: model 1 plus region, ethnicity, marital status, and education level.

Model 3: model 2 plus smoke now status, excessive drinking status, physical activity, oil intake, salt intake, family history of diabetes, BMI.

## Discussion

T2DM is a multi-factorial disease, and various common chronic diseases or pathological status are closely related to initiation of T2DM. Therefore, more attention should be paid to exploring whether multimorbidity of diseases or pathological status could synergistically promote the occurrence of T2DM. To our knowledge, this is the first prospective study to demonstrate the combined effect of MetS and depression on the susceptibility of T2DM from a position of multimorbidity. In this general population-based prospective study of Chinese adults, we uncovered that MetS and depression are independently associated with an increased risk of T2DM. More importantly, the combined exposure of MetS and depression was more strongly related to the risk of T2DM when compared with exposure to a single disease. It is noteworthy that a growing risk of T2DM with MetS combined with depression was more apparent in the population of age ≥60 years, male, and overweight.

Several cohort studies have shown that MetS was associated with an increased risk of T2DM, further verified among the southwest China general population presented in our study. Moreover, a study showed that the MetS is associated with a 5-fold increased risk for incident T2DM (32). The risk of DM with MetS at baseline was twice that of non-MetS, as evidenced by a 10-year follow-up study (33). Based on the Guizhou general population study, we determined that MetS increased 45% the risk of T2DM. However, the detailed underlying mechanisms responsible for the positive correlation between MetS and T2DM are mainly unknown. However, several potential biological mechanisms may partially explain these founds. First, obesity and insulin resistance are commonly co-occurrences in MetS patients (32). Obesity leads to fat accumulation associated with insulin resistance and T2DM (34). Insulin resistance, a key component of MetS, is present in many metabolic disorders, such as T2DM, and is responsible for many metabolic perturbations. Second, MetS activates intracellular pro-inflammatory pathways, promoting systemic inflammatory response to T2DM in impaired metabolic status (35, 36). Furthermore, MetS and T2DM share many common risk factors, including age, obesity, nutrition, and lifestyle modification (32, 37–39).

Similarly, previous studies have shown that depression also increased the risk of T2DM. Luo et al. found that depressive symptoms present as a risk factor for DM among older people (8). Moreover, a prospective study evaluated the correlation between severe depressive episodes and T2DM in China, which is in line with our research. Pathophysiological mechanisms by which depression increased the risk of T2DM also have been explained (40). First, depression was related to hyperactivity of the hypothalamic-pituitary-adrenal (HPA) axis and the sympathetic nervous system (40). This contributed to the increased release of counterregulatory hormones, resulting in abdominal adiposity and insulin resistance (40, 41). Second, the dysregulated immune system functions as a mediator mechanism between depression and increased risk of T2DM. Furthermore, increased C reactive protein, TNF-α, and pro-inflammatory cytokines are associated with depression and T2DM (42, 43). Collectively, the above biological mechanisms may be responsible for the depression-related increased risk of T2DM.

Due to the improvement in lifestyle and increasing social stress, the probability of people simultaneously suffering from metabolic disorders and mental illness has dramatically increased. Our results revealed that MetS combined with depression could be synergistically associated with an increased risk of T2DM, which is higher than that of MetS only or depression only, suggesting that MetS and depression may have a superimposed effect on the occurrence of T2DM. However, the underlying mechanisms for the combined impact of T2DM need to be further determined. It is well-established that multiple organ damage is more likely to increase the risk of various complications than single organ damage. Another plausible reason is that both MetS and depression could induce systemic pro-inflammatory responses, which was a key feature of T2DM, as the action mode of mechanistic pathways underlying the relationships of depression or MetS with the risk of T2DM are similar (44). Therefore, people who suffer from both depression and MetS could generate more severe inflammatory reactions. The multiplicative effect of both depression and MetS might contribute to the substantially more substantial pro-pathogenic effect of the multimorbidity of depression and MetS on T2DM risk. Preventive measures for MetS and depression include a healthy diet, regular exercise, maintaining a healthy weight, and avoiding smoking and excessive alcohol; early detection and treatment are essential (45–47). Our findings imply that individuals with MetS combined with depression should be more severely targeted for preventing and screening T2DM.

In the stratified analysis, we found that patients with depression and Mets are more likely to suffer from T2DM among the population age ≥ 60 years, male, and overweight, and the above results were consistent with previous studies (32, 37, 48, 49). Our results suggested that people with a BMI ≥24 kg/m^2^ and abnormal metabolic rate should be taken seriously in China to prevent and delay the occurrence of T2DM. Hence, adopting a healthy lifestyle pattern and weight loss are a significant determinant of maximizing effectiveness in decreasing the risk of T2DM.

The strengths of our study were its long follow-up duration and prospective cohort study design, which was a prospective study of the impact of depression combined with MetS on the incidence of T2DM. However, our study has several potential limitations. First of all, although we excluded patients with T2DM at baseline, we cannot conclude whether T2DM does cause people with depression at baseline because of a bidirectional relationship between T2DM and depression, which may cause some deviation. Second, some participants were lost to follow-up, and some information regarding confounders was missing. However, sufficient events and a high follow-up rate provided adequate statistical power. Finally, the enrolled participants were only restricted to Guizhou Province, China. So, the extrapolation of the results should be cautious. Therefore, prospective large-scale studies are needed to verify these results in other regional populations.

## Conclusions

In conclusion, this study indicated depression and MetS are associated with increased T2DM. People who simultaneously have depression and MetS have an apparent higher risk of T2DM than those with depression or metabolic syndrome alone. Our results highlight that the multimorbidity of metabolic disorder and psychological disorder is more suffering from T2DM. Therefore, it is meaningful to prevent and effectively treat metabolic disorders and mental problems, especially for MetS and depression, to improve current health and reduce the risk of future T2DM. Our study provides additive value for preventing the development of T2DM from the position of prevention and control multimorbidity. Additional studies or randomized control trials are needed to confirm our conclusions and to examine further underlying mechanisms for the multimorbidity of depression and MetS with enhanced risk of T2DM.

## Data availability statement

The original contributions presented in the study are included in the article/supplementary material. Further inquiries can be directed to the corresponding authors.

## Ethics statement

The studies involving humans were approved by Institutional Review Committee of Guizhou Center for Disease Control and Prevention (No.S2017-02). The studies were conducted in accordance with the local legislation and institutional requirements. The participants provided their written informed consent to participate in this study. Written informed consent was obtained from the individual(s) for the publication of any potentially identifiable images or data included in this article.

## Author contributions

YC: Conceptualization, Formal analysis, Project administration, Writing – original draft, Writing – review & editing, Methodology, Visualization. SZ: Data curation, Methodology, Writing – review & editing. SF: Data curation, Writing – review & editing. YY: Data curation, Methodology, Writing – review & editing. KT: Methodology, Writing – review & editing. LL: Writing – review & editing. RD: Writing – review & editing. XD: Writing – review & editing. YW: Writing – review & editing. MZ: Conceptualization, Writing – review & editing, Formal analysis. TL: Conceptualization, Writing – review & editing, Formal analysis, Funding acquisition.
